# How does centralized isolation treatment strategy affect the medical staff's mental health during the COVID-19 pandemic?

**DOI:** 10.3389/fpubh.2022.1055564

**Published:** 2023-01-04

**Authors:** Wei Song, Dongmei Huang, Jiejing Yu

**Affiliations:** ^1^Yatai School of Business Management, Jilin University of Finance and Economics, Changchun, China; ^2^Department of Psychology, School of Philosophy and Sociology, Jilin University, Changchun, China

**Keywords:** COVID-19, medical staff, centralized isolation treatment strategy, mental health, mobile cabin hospitals

## Abstract

**Background:**

During the coronavirus 2019 (COVID-19) pandemic, the Chinese Government adopted a centralized isolation treatment (CIT) strategy for patients, which has greatly improved the efficiency of the pandemic response. However, compared to those in local hospitals, anti-COVID-19 medical staff in mobile cabin hospitals, where the CIT strategy was adopted, suffered more mental health problems. This study aimed to explore how the CIT strategy affected the medical staff's mental health by comparing anti-COVID-19 medical staff who worked in mobile cabin hospitals to those in fever clinics of local hospitals.

**Methods:**

Following the standard scale development procedure, this study first developed a scale measuring the mental health of anti-COVID-19 medical staff. Using SPSS 23.0 and Amos 23.0 software, the exploratory factor analysis (EFA), confirmatory factor analysis (CFA), and reliability analysis method were conducted to support the scale development. In the main investigation, a survey method using the developed scale was used, and 839 anti-COVID-19 medical staff from five hospitals in northern China were recruited as participants by snowball-sampling method. The first survey was conducted in February 2020, when the first round of COVID-19 was at a serious time. In April 2020, after the first round of COVID-19 in China was initially contained, and medical staff who worked in mobile cabin hospitals returned to local hospitals, a follow-up survey was conducted on these participants. Using SPSS 23.0 software, a series of 2 × 2 mixed-design ANOVA was conducted, in which working conditions (mobile cabin hospital vs. local hospital) served as a between-subject factor, time points (during vs. after the first round of COVID-19) served as a within-subject variable, and the indicators of the medical staff's mental health served as dependent variables respectively.

**Results:**

The reliability and validity of the developed scale were desirable. The mental health problems of anti-COVID-19 medical staff were mainly manifested as anxiety, powerlessness, fear of infection, and somatization. Compared to those who worked in local hospitals, anti-COVID-19 medical staff who worked in mobile cabin hospitals where the CIT strategy was adopted suffered more powerlessness, fear of infection, and somatization. After returning to local hospitals, symptoms of fear of infection and powerlessness of medical staff who used to work in mobile cabin hospitals decreased significantly. However, their anxiety symptoms were not relieved, and their somatization symptoms even increased.

**Conclusion:**

This study implied that the mental health of anti-COVID-19 medical staff in mobile cabin hospitals adopting CIT was worse than in local hospitals. Moreover, with the first outbreak in remission, the mental health recovery of medical staff in CIT hospitals was slower than in local hospitals. Relevant practitioners should pay more attention to the mental health condition of anti-COVID-19 medical staff who work in CIT hospitals. The psychological assistance service for them should continue even after they return to the local hospitals.

## Introduction

Since the coronavirus 2019 (COVID-19) outbreak, the Chinese Government has adopted a strategy of centralized isolation treatment (CIT) for patients. Mobile cabin hospitals, such as Fire God Mountain and Thunder God Mountain hospitals in Wuhan, were established for the centralized treatment of patients with novel coronavirus pneumonia. Compared to local hospitals, these hospitals are only used to treat patients with COVID-19 and are equipped with experienced medical staff and equipment according to the characteristics of COVID-19. The CIT strategy has many advantages, such as avoiding cross-infection of patients, optimizing resource allocation, and improving treatment efficiency (1–3). However, its potential negative effects on the medical staff's mental health have not been explored sufficiently.

Previous studies have found that anti-COVID-19 medical staff (i.e., the doctors and nurses specially assigned to treat COVID-19 patients) suffer from mental health problems, such as anxiety, depression, and posttraumatic stress disorder (4, 5). However, as for the affecting factors, the existing literature mainly focuses on the personal level, such as the demographic characteristics of medical staff or the social support they receive (6, 7). Few studies have paid attention to the affecting factors on the systematic level, such as medical strategies that the government adopted. Although the CIT strategy has been proven effective in response to the COVID-19 pandemic (8), it may bring more severe challenges to medical staff than those in local hospitals. For example, they face a higher risk of virus exposure (9), which may make them live in anxiety and fear of infection (10). The higher work overload may not only bring them physical fatigue but also threaten their mental health (11, 12). The increasing number of deaths and the shortage of medical resources may also bring more sense of frustration and powerlessness, leading to moral injury (13).

Existing studies on the mental health of frontline anti-COVID-19 medical staff mainly regard them as an undifferentiated group (4, 14, 15) but neglect the potential differences between their working conditions. In the early stage of the COVID-19 outbreak in 2020, due to the limited resources, China temporarily adopted a parallel treatment strategy. Some anti-COVID-19 medical staff were sent to work in CIT hospitals, whereas some stayed in fever clinics of local hospitals. Although both groups took on the task of receiving and treating patients with COVID-19, they worked under different treatment strategies and working conditions. However, how these two working conditions affect medical staff's mental health and how long such effects will last have not been revealed in the existing literature.

In view of the above background, this study aims to solve the following research problems: first, whether CIT strategy will bring more serious negative impact on the mental health of medical staff; Second, will the impact continue after medical staff leave the CIT working environment? To answer these questions, this study first conducted a survey at the beginning of the first outbreak period in China in February 2020, and compared the mental health of medical staff in CIT hospitals to those who worked in fever clinics of local hospitals. In April 2020, after the first round of COVID-19 in China was initially contained, and medical staff who worked in mobile cabin hospitals returned to local hospitals, a follow-up survey was conducted on these participants. Then, we compared their mental health condition between these two time points. Thus, working condition (CIT hospitals vs. local hospitals) and time point (during vs. after the first round of COVID-19) served as the independent variables of this study, and the mental health indicators of anti-COVID-19 medical staff served as the dependent variables.

This study was carried out from February to April 2020, when the COVID-19 pandemic was in its first round of outbreak in China, and other countries had not yet experienced a large-scale outbreak. Faced with the unprecedented situation and the surge in the number of patients, the Chinese government's response to the pandemic and the treatment strategy for patients were exploratory, and CIT strategy was one of them. At such a time and place, the necessity and practical purpose of current study are mainly shown in the following aspects: first, revealing the negative impact of CIT strategy on the mental health of medical staff can remind interested parties to pay more attention to this problem; Second, comparing the mental health status of anti-COVID-19 medical staff under different working conditions is conducive to clarify the key groups of mental health care. Third, describing the characteristics of the medical staff's mental health at different pandemic development stages will help providing mental health services for them more accurately at different time points. Therefore, this study can provide reference for more accurate practical guidance on mental health care for medical staff in the future public health emergencies.

## Methods

### Scale development

As anti-COVID-19 medical staff are faced with unique working conditions, their stress response patterns and mental health problems also have some particularities. Therefore, this study developed the Mental Health Scale for Anti-COVID-19 Medical Staff based on the uniqueness of their mental health symptoms. The development procedure of the scale is as follows:

#### Step 1: Item construction

In the early stage of the COVID-19 outbreak in February 2020, the authors took part in the psychological assistance team providing psychological counseling services for anti-COVID-19 medical staff. In the process of psychological assistance, the team members recorded the mental health symptoms that anti-COVID-19 medical staff often feel during work from 186 counseling cases. Two doctoral students in psychology summarized the records and merged the ones with similar meanings. In these records, 23 symptoms were mentioned by at least 50% of the medical staff in consultation. Then, we compiled these 23 items into questionnaire items as the original pool of questions (see Table 1). In the questionnaire, participants were asked to report the frequency they encountered the 23 symptoms recently on five-point scales (1 = not at all, 5 = very frequently).

**Table 1:** Demographic characteristics of the participants.

**Variables**	**Levels**	***N***	**%**
Working conditions	Mobile cabin hospital	266	31.7
	Local hospital	573	68.3
Gender	Male	136	16.3
	Female	703	83.8
Age, years	< 20	13	1.5
	20–29	177	21.1
	30–39	434	51.7
	40–49	158	18.8
	>50	56	6.8
Position	Doctor	235	28
	Nurse	598	71.3
	Manager	6	0.7

#### Step 2: Preinvestigation

Although we had compiled the original question pool describing the mental health symptoms of anti-COVID-19 medical staff in the previous step, it is not clear which dimensions these symptoms can belong to, that is, the structure of the scale has not been determined. To explore the structure of the scale and further select the items for formal questionnaire based on the quantitative criteria, a preinvestigation was conducted. In preinvestigation, the original questionnaire with 23 items was sent out to 675 anti-COVID-19 medical staff recruited online (female = 83.4%, *M*_age_ = 30.79 ± 8.28). After the questionnaires were collected, the exploratory factor analysis (EFA) method was used for the 23 initial items to explore the structure of the scale. Then, based on the results of EFA, we further selected 17 final items from the question pool of 23 items to form the formal version of the questionnaire (see details in the section of Results).

#### Step 3: Reinvestigation

After the formal questionnaire with 17 items was determined according to the results of the preinvestigation, we further conducted a systematic reliability and validity analysis for it. In order to ensure the robustness of the results, we obtained another sample through reinvestigation. In the reinvestigation, the formal questionnaire consisting of 17 items in four factors was sent to 454 anti-COVID-19 medical staff recruited online (female = 82.6%, *M*_age_ = 31.12 ± 8.05). After the questionnaires were collected, confirmatory factor analysis (CFA) and reliability analysis methods were used to check the reliability and validity of the formal questionnaire (see details in the section of Results).

### Main investigation

Using the scale compiled by the above procedures, the main investigation on the mental health of anti-COVID-19 medical staff was conducted. The authors contacted the managers of five hospitals in northern China and distributed questionnaires to anti-COVID-19 medical staff through snowball-sampling in these five hospitals. A total of 839 anti-COVID-19 medical staff in China were recruited as participants. The demographic characteristics of these participants are listed in Table 1. Among these 839 anti-COVID-19 medical staff, 266 participants were sent to mobile cabin hospitals for support, where the CIT strategy was adopted, and 573 participants stayed in fever clinics of local hospitals to treat COVID-19 patients. Therefore, the effects of the CIT strategy were analyzed by comparing the mental health levels of the two groups of participants. The first survey was conducted in February 2020 when the first round of COVID-19 was at a serious time. In April 2020, after the first round of COVID-19 in China was initially contained, a follow-up survey was conducted on these participants. At this time, mobile cabin hospitals had been closed due to reduced cases, and medical staff who worked in mobile cabin hospitals had returned to local hospitals. Therefore, the subsequent effects of the CIT strategy were analyzed by comparing the mental health levels of the participants between these two time points.

### Statistical analysis

In the section of scale development, using the data collected from preinvestigation and reinvestigation, we conducted EFA and reliability analysis in SPSS 23.0 and conducted CFA in Amos 23.0. In the section of main investigation, we first calculated the average scores of each participant on the four factors (i.e., anxiety, powerlessness, fear of infection, and somatization) as the corresponding indicators of their mental health symptoms. The higher the score, the more serious the mental health symptoms. Then, a series of 2 × 2 mixed-design analysis of variance (ANOVA) was conducted, in which working conditions (mobile cabin hospital vs. local hospital) served as a between-subject factor, time points (during vs. after the first round of COVID-19) served as a within-subject variable, and the four indicators of the medical staff's mental health served as dependent variables respectively.

## Results

### Results of scale development

After the formal questionnaire was formed, we invited two professors of health psychology to evaluate the validity of the questionnaire using qualitative methods, and they agreed that the questionnaire had desirable face validity and content validity. In addition to the qualitative evaluation, we also adopt the following quantitative methods to analyze the reliability and validity of the questionnaire.

#### Results of EFA in preinvestigation

First, Kaiser-Meyer-Olkin test (*KMO* value = 0.963) and Bartlett sphericity test [$\chi_{\left(253\right)}^{2}$ = 14,809.28, *p* < 0.001] verified that the sample was suitable for EFA. Then, principal component analysis and the maximum variance rotation method were used in EFA to determine the final factors. Results revealed that four factors were extracted with the criteria of eigenvalues over 1. The rotated component matrix is shown in Table 2. Items 6, 7, 18, and 23 were deleted because their loadings on any factor were < 0.65. Items 12 and 17 were deleted because of cross-loading (i.e., although their loadings on one factor were > 0.65, their loadings on another factor were > 0.4). Based on the content analysis of the remaining 17 items, the four factors were named anxiety (including Item 1–5), powerlessness (including Item 8–11), fear of infection (including Item 13–16), and somatization (including Item 19–22).

**Table 2:** Items and factor loadings based on EFA.

**Items**	**Factor loadings**			
	**Factor 1**	**Factor 2**	**Factor 3**	**Factor 4**
1. Spontaneous heart palpitations	**0.828**	0.275	0.222	0.251
2. Feeling restless and unsettled	**0.826**	0.249	0.239	0.269
3. Feeling nervous easily	**0.812**	0.264	0.228	0.261
4. Feeling of unexplained fear or panic	**0.805**	0.302	0.300	0.201
5. Unable to concentrate	**0.712**	0.309	0.215	0.298
6. Thinking about death a lot (D)	0.642	0.428	0.307	0.185
7. Feeling powerless in the face of a dying patient (D)	0.531	0.447	0.329	0.172
8. It pains me to see the patient helpless	0.272	**0.818**	0.191	0.205
9. It is sad to see the death of patients	0.220	**0.776**	0.280	0.208
10. I was devastated by the death of young patients	0.209	**0.762**	0.259	0.153
11. Guilt about the patient who died	0.346	**0.753**	0.207	0.203
12. Despair in the face of increasing patients (D)	0.447	0.686	0.229	0.170
13. Checking frequently for fear of lax self-protection	0.165	0.260	**0.780**	0.295
14. Fear of infection due to poor immunity	0.320	0.311	**0.745**	0.262
15. Monitoring body temperature frequently	0.249	0.155	**0.742**	0.228
16. Feeling sensitive and insecure about being infected	0.233	0.246	**0.742**	0.261
17. Fear of being infected by patients during treatment (D)	0.309	0.404	0.677	0.246
18. There are images that cannot be erased in my mind (D)	0.462	0.185	0.548	0.290
19. Dizziness and headache	0.186	0.137	0.217	**0.820**
20. Body pain (e.g., chest pain and lower back pain)	0.310	0.141	0.284	**0.756**
21. Many dreams and easy to wake up	0.236	0.331	0.273	**0.714**
22. Insomnia and difficulty falling asleep	0.261	0.292	0.317	**0.687**
23. Feel heavy in the limbs (D)	0.461	0.138	0.316	0.531
Variance contribution	23.404%	18.978%	18.265%	15.230%

D means the items were deleted because they did not meet the criteria. The bold values mean they meet the criteria.

#### Results of CFA and reliability analysis in reinvestigation

SPSS version 24.0 was used to conduct reliability analysis, and Amos 24.0 was used to conduct CFA. In Table 3, the factor loadings of the 17 items on the four factors were all > 0.7, the composite reliability values and Cronbach's α of each four factors were all higher than the criterion of 0.7, and the values of average variance extracted (AVE) of each four factors were all higher than the criterion of 0.5. The model fits of the CFA were also desirable. These results suggested that the reliability and validity of the revised scale are good.

**Table 3:** Results of reliability and validity analysis.

**Factor**	**Item**	**Factor loading**	**AVE**	**Composite reliability**	**Cronbach's α**	**Model fit**
Anxiety (A)	A1	0.784	0.813	0.956	0.957	χ^2^/*df* = 3.33, GFI = 0.875, CFI = 0.948, NFI = 0.927, TLI = 0.937, RMR = 0.046, RMSEA = 0.084
	A2	0.930				
	A3	0.926				
	A4	0.921				
	A5	0.939				
Powerlessness (P)	P1	0.844	0.695	0.901	0.906	
	P2	0.761				
	P3	0.863				
	P4	0.862				
Fear of infection (FI)	FI1	0.911	0.730	0.915	0.894	
	FI2	0.865				
	FI3	0.747				
	FI4	0.776				
Somatization (S)	S1	0.815	0.632	0.873	0.869	
	S2	0.831				
	S3	0.778				
	S4	0.754				

### Results of main investigation

#### Effects on anxiety

**Figure 1:**
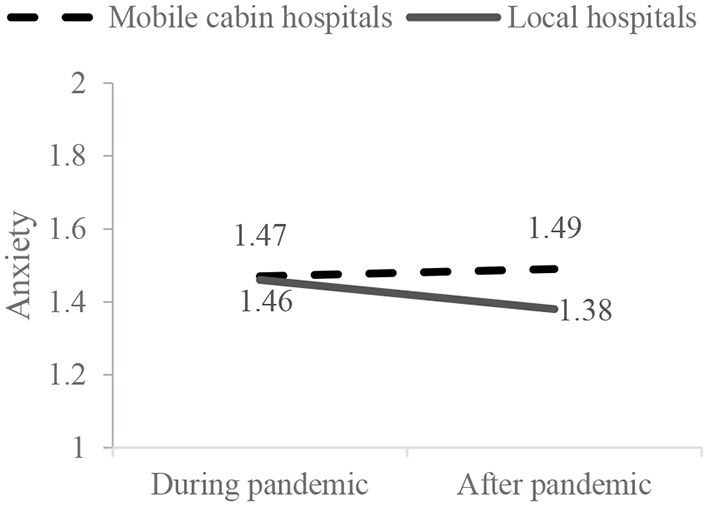
Interactive effects on the medical staff's anxiety.

Results of mixed-design ANOVA showed that the interactive effect of working conditions and time points on medical staff's anxiety were significant (*F* = 7.63, *p* = 0.006). Simple-effect analyses were further conducted (Figure 1). During the first round of COVID-19, there was no significant difference between the anxiety of medical staff in mobile cabin hospitals and local hospitals (*F* = 0.00, *p* = 0.958). After the first round of COVID-19, the anxiety of medical staff in local hospitals was significantly lower than those in mobile cabin hospitals (*F* =3.67, *p* = 0.056). The anxiety of medical staff in local hospitals was significantly reduced after the first round of COVID-19 (*F* = 16.69, *p* < 0.001). However, the anxiety of medical staff in mobile cabin hospitals was not reduced after the first round of COVID-19 (*F* = 0.31, *p* = 0.576). These results suggested that, during the first round of COVID-19, medical staff in mobile cabin hospitals and local hospitals suffered the same level of anxiety. However, with the control of the first round of the pandemic, the anxiety of medical staff in local hospitals was significantly alleviated, whereas medical staff who worked in mobile cabin hospitals maintained a high level of anxiety after they had returned to local hospitals.

#### Effects on powerlessness

**Figure 2:**
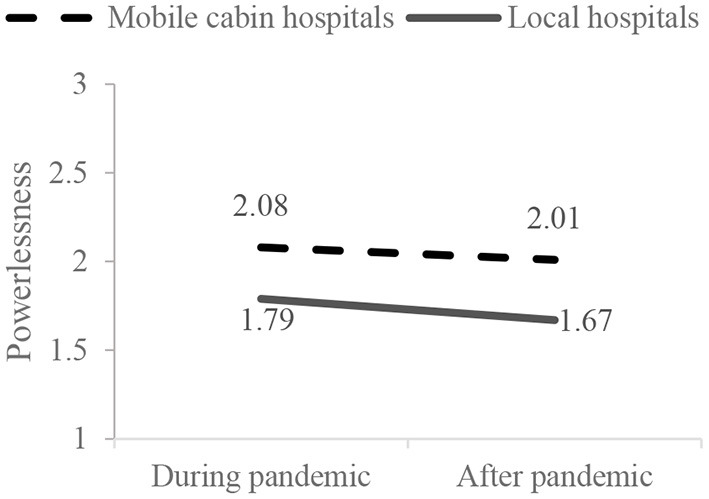
Interactive effects on the medical staff's powerlessness.

Results of mixed-design ANOVA showed that the interactive effect of working conditions and time points on medical staff's powerlessness were significant (*F* = 7.63, *p* = 0.044). Simple-effect analyses were further conducted (Figure 2). During the first round of COVID-19, the powerlessness of medical staff in mobile cabin hospitals was higher than in local hospitals (*F* = 16.48, *p* < 0.001). After the first round of COVID-19, the powerlessness of medical staff in mobile cabin hospitals was also significantly higher than in local hospitals (*F* =26.382, *p* < 0.001). The powerlessness of medical staff in local hospitals was significantly reduced after the first round of COVID-19 (*F* = 56.00, *p* < 0.001). The powerlessness of medical staff in mobile cabin hospitals was also reduced after the first round of COVID-19 (*F* = 7.83, *p* = 0.005). These results suggested that, during the first round of COVID-19, medical staff in mobile cabin hospitals suffered a higher level of powerlessness than those in local hospitals. Although the powerlessness of medical staff in cabin hospitals and local hospitals was significantly alleviated with the control of the first round of the pandemic, the relief of medical staff's powerlessness in local hospitals was more dramatic than that of medical staff who had worked in mobile cabin hospitals.

#### Effects on fear of infection

**Figure 3:**
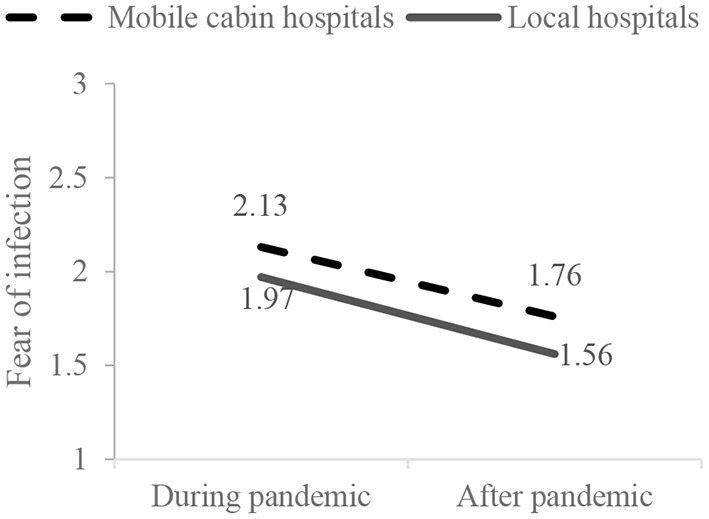
Interactive effects on the medical staff's fear of infection.

Results of mixed-design ANOVA showed that the main effect of working conditions on medical staff's fear of infection was significant (*F* = 8.91, *p* = 0.003), suggesting that medical staff in mobile cabin hospitals suffered a higher level of fear of infection than those in local hospitals. The main effect of time points was also significant (*F* = 270.95, *p* < 0.001), suggesting that the fear of infection of medical staff was reduced after the first round of COVID-19. The interactive effects of working conditions and time points were nonsignificant (*F* = 0.69, *p* = 0.406; Figure 3), suggesting no difference in the decreasing trend of fear of infection between medical staff in local hospitals and mobile cabin hospitals.

#### Effects on somatization

**Figure 4:**
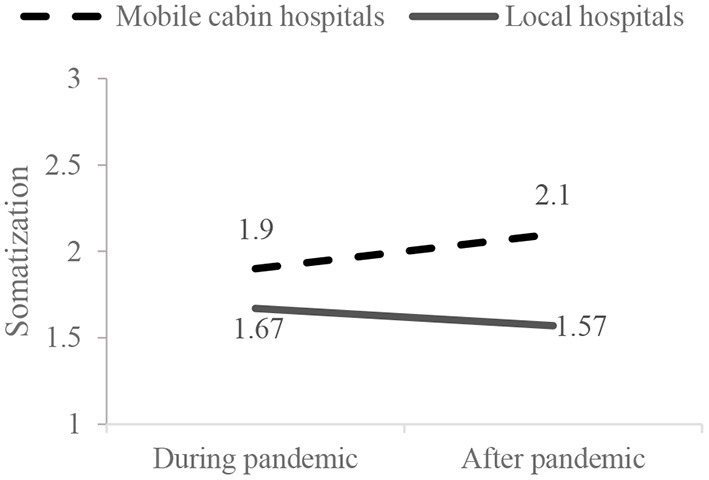
Interactive effects on the medical staff's anxiety.

Results of mixed-design ANOVA showed that the interactive effect of working conditions and time points on medical staff's somatization were significant (*F* = 46.75, *p* < 0.001). Simple-effect analyses were further conducted (Figure 4). During the first round of COVID-19, the somatization of medical staff in mobile cabin hospitals was higher than in local hospitals (*F* = 12.95, *p* < 0.001). After the first round of COVID-19, the somatization of medical staff in mobile cabin hospitals was significantly higher than in local hospitals (*F* = 73.15, *p* < 0.001). The somatization of medical staff in local hospitals was significantly reduced after the first round of COVID-19 (*F* = 15.95, *p* < 0.001). In contrast, the somatization of medical staff in mobile cabin hospitals even increased after the first round of COVID-19 (*F* = 30.83, *p* = 0.005). These results suggested that, during the first round of COVID-19, medical staff in mobile cabin hospitals suffered a higher level of somatization than those in local hospitals. However, with the control of the first round of the pandemic, the somatization of medical staff in local hospitals was significantly alleviated. In contrast, the somatization of medical staff who had worked in mobile cabin hospitals was even more serious after they had returned to local hospitals.

## Discussion

Using the EFA method, this study found that the mental health problems of anti-COVID-19 medical staff mainly manifest as anxiety, powerlessness, fear of infection, and somatization. By comparing with the existing literature, it can be found that anxiety (16–19), fear (18, 20, 21), and somatization (17, 18, 22) are mental health problems commonly encountered by anti-COVID-19 medical staff worldwide, such as Netherlands (23), Pakistan (24), and Mexico (21). These findings are consistent with the conclusions of ours.

Although there has been literature on the powerlessness experienced by residents in COVID-19 pandemic (25–27), few studies have focused on this symptom of anti-COVID-19 medical staff. Our study found that powerlessness is also a symptom frequently reported by anti-COVID-19 medical staff when seeking psychological assistance services. It is specifically manifested in the sense of guilt, pain, sadness and devastated when seeing patients die during the treatment process. This may be because the data of this study were collected at the very beginning of the COVID-19 outbreak (February to April 2022), and the participants in the study were the first batch of anti-COVID-19 medical staff who worked on the front lines. At that time, on the one hand, the death rate of COVID-19 disease was high, and on the other hand, the medical staff were not fully prepared for the severity and cruelty of the disease, which may cause the sense of powerlessness to become a prominent symptom they experienced. This conclusion reminds us that it is very important to pay more attention to the powerlessness of front-line medical staff at the beginning of public health emergency. However, in the following time, the death rate of COVID-19 disease has gradually decreased, and the knowledge and preparation of medical staff have also been constantly improved. The symptom of powerlessness may continue to decline or turn into other long-term effects on mental health, such as post-traumatic stress disorder (PTSD) (5, 28).

There are still some mental health symptoms in the existing literature that have not been revealed in our study, such as depression (6, 16), PTSD (5, 28), and burn out (12, 29). This may also be because our study was carried out in the first 2 months of the outbreak. In this period, the psychological symptoms of medical staff may be more likely derived from the stress response to the situation, such as anxiety about the unpredictable situations, fear of infection, feeling powerless about the patient's death, and somatization caused by high intensity work. Over time and as the pandemic progresses, these situational symptoms may convert into more long-term mental health problems, such as depression, PTSD, and burn out. This conclusion may implies that the mental health problems experienced by medical staff may be different at different stages of public health emergencies. This could be an interesting direction for future research.

Although previous literature revealed the differences in mental health among the potential groups of anti-COVID-19 medical staff, such as differences between frontline and nonfrontline (30–32) and differences between different posts (33), this study found that such difference was also manifested in medical staff under different working conditions. Compared to those who worked in local hospitals, anti-COVID-19 medical staff who worked in mobile cabin hospitals where the CIT strategy was adopted suffered more powerlessness, fear of infection, and somatization. This may be because medical staff in mobile cabin hospitals suffered a higher risk of virus exposure and a higher work overload. These results suggested that although the CIT strategy has high efficiency in coping with COVID-19, it also brings more pressure and mental health problems to medical staff.

An important contribution of this study is the analysis of the subsequent effect of the CIT strategy. After returning to local hospitals, medical staff who used to work in mobile cabin hospitals have significantly alleviated the fear of infection and powerlessness. This may be because after the pandemic is eased and they leave the high-risk and high-intensity working condition in mobile cabin hospitals, the medical staff's fear of infection was reduced, and their sense of power was restored. However, the anxiety symptoms of medical staff were not relieved after their return to local hospitals. This may imply that the effects of work experience in mobile cabin hospitals on their anxiety may be long-term rather than situational. Moreover, the somatization symptoms of medical staff who worked in mobile cabin hospitals even increased after they had returned to local hospitals. This may be because medical staff are in a highly stressed state of mind and body when they work in mobile cabin hospitals; when they get rest, the negative emotions accumulated previously may gradually manifest in a somatic way. Such conclusions inspire researchers to pay more attention to anxiety and somatization symptoms when providing psychological assistance to medical staff who have experienced working in mobile cabin hospitals.

Based on the above conclusions, we provide the following suggestions to relevant practitioners: first, the mental health symptoms experienced by medical staff at the initial stage of a public health emergency may be different from those at the later stage. According to our findings, practitioners should pay more attention to the symptoms of anxiety, powerlessness, fear, and somatization at the beginning of public health emergencies. Secondly, relevant departments should provide more psychological care for medical staff working in mobile cabin hospitals, even after they return to the local hospital. This is because, according to our investigation results, although some symptoms of these medical staff have improved after leaving the mobile cabin hospitals (e.g., fear of infection and powerlessness), some symptoms have not (e.g., anxiety), or even increased (e.g., somatization).

Many studies have explored the factors that affect the mental health of anti-COVID-19 medical staff (6, 7). This study found that treatment strategies and work environment are also important factors. In the future research, it is an important research issue to explore how to effectively protect and promote the mental health of medical staff based on these factors. In addition to directly providing psychological services for medical staff, existing studies have also found that ways such as optimizing the working environment, adopting scientific management methods, and strengthening training can improve the wellbeing and mental health of medical staff (34–38). Then, how to integrate these methods into hospital management in the context of public health emergencies to indirectly promote the mental health of medical staff can also be an important direction of future research.

## Data availability statement

The raw data supporting the conclusions of this article will be made available by the authors, without undue reservation.

## Ethics statement

The studies involving human participants were reviewed and approved by Jilin University. Written informed consent for participation was not required for this study in accordance with the national legislation and the institutional requirements.

## Author contributions

WS and DH were involved in the conceptualization, methodology, and investigation referred to in this paper. DH provided leadership to the team, was responsible for revising the article, and revised the manuscript. JY conducted the data analysis. WS wrote the first draft of the paper. All authors provided a written contribution and approved the final version.
